# Alveolar recruitment maneuvers in acute lung injury/acute respiratory distress syndrome

**DOI:** 10.4103/0972-5229.53107

**Published:** 2009

**Authors:** Jose Chacko, Usha Rani

**Affiliations:** **From:** Jose Chacko, Multidisciplinary Intensive Care Unit, Manipal Hospital, Bangalore, India; 1 Respiratory Therapist, Manipal Hospital, Bangalore, India

**Keywords:** Acute lung injury, acute respiratory distress syndrome, recruitment maneuvers

## Abstract

Mechanical ventilation can worsen lung damage in acute lung injury and acute respiratory distress syndrome. The use of low tidal volumes is one of the strategies that has been shown to reduce lung injury and improve outcomes in this situation. However, low tidal volumes may lead to alveolar derecruitment and worsening of hypoxia. Recruitment maneuvers along with positive end-expiratory pressure may help to prevent derecruitment. Although recruitment maneuvers have been shown to improve oxygenation, improved clinical outcomes have not been demonstrated. The optimal recruitment strategy and the type of patients who might benefit are also unclear. This review summarizes the impact of recruitment maneuvers on lung mechanics and physiology, techniques of application, and the clinical situations in which they may be useful.

## Introduction

Injurious ventilation strategies can worsen lung damage in Acute Respiratory Distress Syndrome (ARDS).[1] During positive pressure ventilation, there are alveolar units in varying degrees of collapse adjacent to areas of fully open alveoli. When inflated, shear stresses develop between open and closed alveoli that may be injurious.[2] Cyclical opening and closure of alveolar units can also result in the generation of shear forces.[3] Both these mechanisms can cause endothelial and epithelial injury leading to loss of integrity of the alveolar capillary membrane, bacterial translocation from the lung, and cellular inflammatory damage.[4,5] Shear forces also lead to the activation of nuclear factor kappa B that results in the expression of cytokines, resulting in the initiation and propagation of multiorgan dysfunction syndrome.[6] There is strong evidence that low tidal volume ventilatory strategies improve survival.[7] However, the use of small tidal volumes can cause alveolar derecruitment and arterial hypoxemia. In the ARDSNet trial, patients randomized to the low tidal volume group had lower PO_2_ levels, possibly due to alveolar derecruitment.[7] Strategies to recruit closed lung units and prevent derecruitment might be beneficial in improving gas exchange as well as reduce the incidence of ventilator-induced lung injury.

## Effects of recruitment maneuvers

Although ARDS is described as a diffuse disease process affecting both the lungs, CT scan based studies have revealed a nonuniform pattern of involvement.[8] There are regions within the lung that are collapsed or consolidated, mainly confined to the dependent areas, and other regions which are open and ventilate in a relatively normal fashion.[9] The considerably reduced portion of the lung that is amenable to ventilation has been referred to as the ‘baby lung’.[10] Injurious ventilatory strategies might aggravate damage to the lung. High peak pressures and high tidal volumes have been shown to be associated with a significantly higher incidence of barotrauma.[11] Mechanical ventilation using high tidal volumes also results in the release of inflammatory mediators such as interleukins 1 and 6, tumour necrosis factor alpha, etc, that may be attenuated by optimizing ventilator strategy.[12] Ventilation with low tidal volumes below the lower inflexion point might also result in progression of lung injury.[3] Thus, there needs to be a careful balance between strategies that use excessive pressures and volumes that might aggravate lung injury and the use of low tidal volume strategies that might lead to alveolar derecruitment and significant hypoxemia. It may be possible to open up or ‘recruit’ at least some of the collapsed areas by the use of continuous or repetitive application of increased levels of distending alveolar pressure, much higher than that recommended for the ventilation of patients with Acute Lung Injury (ALI). The applied pressure as well as the duration of its application is likely to determine the effectiveness of a Recruitment Maneuver (RM). The efficacy of an RM is likely to be directly related to the number of closed lung units. In other words, such maneuvers will be less effective if most of the lung units are already open. Thus, the potential for recruitment may be greater when lower Positive End-Expiratory Pressure (PEEP) levels are being used compared to higher PEEP levels.[13,14] However, the potential for recruitment may also depend on the number of closed alveolar units and may be predictable using physiological variables including PaO_2_/FiO_2_ (P/F) ratio, PCO_2_ and compliance.[15] It is important to understand that while PEEP can maintain recruitment, intermittent higher pressures would be required to initiate the process. After recruitment has been initiated, it may be possible to maintain it with lower pressures. In fact, alveolar to pleural pressure difference of more than 60cm of H_2_ O may be required to open collapsed lung units.[16]

## Techniques

Several methods have been employed to carry out RMs in the clinical setting as well as in experimental models [Table 1]. Rothen *et al.* showed that a sustained inflation maneuver of 40cm of H_2_ O, for 7–8 seconds might re-expand all collapsed lung units as evident on CT scans of the chest and improve oxygenation in anesthetized subjects.[16] In another study, three consecutive sighs of plateau pressure 45cm of H_2_O were applied every minute for an hour. This resulted in an increase in the end- expiratory lung volume, reduction in the intrapulmonary shunt, and improvement in oxygenation. [17] Lapinsky *et al.* applied a sustained inflation maneuver of 45cm of H_2_O or the peak pressure at a tidal volume of 12mls/kg, whichever was lower, for a period of 20 seconds. An improvement in oxygen saturation was noted in all patients; in 10 out of 14 patients, this was sustained for up to four hours. No significant adverse effects were noted.[18] Lim *et al.* used an ‘extended sigh’ (e-sigh) as an RM.[19] This involved gradually reducing tidal volumes from 8–2mls/ kg and increasing the PEEP from 10–25cm of H_2_O in a stepwise manner, each step lasting 30 seconds. When a tidal volume of 2mls/kg and a PEEP of 25cm of H_2_O were reached, a Continuous Positive Airway Pressure (CPAP) level of 30cm of H_2_O was applied for 30 seconds following which a reverse sequence was applied till the baseline settings were reached. The authors could demonstrate a statistically significant increase in PO_2_ and static compliance with this maneuver. RMs may also be practicable in spontaneously breathing patients. Patroniti *et al.* applied a CPAP of 20% higher than the peak pressure on pressure support ventilation for 3–5seconds every minute, for at least an hour. The authors could demonstrate an improvement in oxygenation and lung compliance with this maneuver.[20] Constantin *et al.* compared two different RMs - (a) CPAP of 40cm of H_2_O for 40 seconds without tidal ventilation and (b) an e-sigh comprising of increasing the PEEP level to 10cm above the lower inflexion point on the pressure–volume curve for a period of 15 minutes, on volume-controlled ventilation. Both maneuvers improved oxygenation at 5 and 60 minutes; there was no statistically significant difference in the degree of improvement. However, only the e-sigh was associated with an increase of recruited volume at 5 and 60 minutes. The systolic pressure dropped below 70mm Hg during the CPAP maneuver on two occasions resulting in interruption of the RM while there was no significant blood pressure drop with the e-sigh.[21] Other studies also suggest that RMs using a sustained high inflation pressure may cause hemodynamic compromise and hence may not be the preferred technique.[22,23] Some level of sedation is usually required to carry out an RM, but muscle paralysis is not absolutely necessary. The patient should be monitored for hypotension and fall in oxygen saturation during the RM. It is also important to emphasize that an RM should be followed up with an appropriate PEEP level, set in a decremental fashion; otherwise derecruitment may occur.[24]

**Table 1:** Studies evaluating different types of recruitment maneuvers

Study	Year of publication	Technique/intervention	Findings
Pelosi *et al.*[17]	1999	Three consecutive sighs of plateau pressure 45 cm of H_2_O every minute for an hour	Increase in the end expiratory lung volume, reduction in the intrapulmonary shunt and improvement in oxygenation
Lapinsky *et al.*[18]	1999	Sustained inflation maneuver of 45 cm of H_2_O or the peak pressure at a tidal volume of 12 mls/kg, whichever was lower, for a period of 20 seconds	Improvement in oxygen saturation in all 14 patients studied; sustained up to four hours in 10 patients. No significant adverse effects noted
Rothen *et al.*[16]	1999	Sustained inflation of 40 cm of H_2_O for 26 seconds in anesthetized patients	Inflation of the lungs to a pressure of 40 cm H_2_O, maintained for 7–8 seconds only, may re-expand all previously collapsed lung tissue, as detected by lung computed tomography, and improve oxygenation
Lim *et al.*[19]	2001	On volume controlled mode, Vt (tidal volume) reduced by 2 mls/kg and PEEP increased by 5 cm of H_2_O every 30 seconds. At Vt 2 mls/kg and PEEP 25, CPAP 30 cm of H_2_O was applied for 30 seconds. Basal settings reached in reverse sequence	Increase in PO_2_ and static compliance, sustained for the duration of the study. No major respiratory or hemodynamic complications
Bein *et al.*[41]	2002	Progressive increase in peak airway pressure (within 30 sec) up to 60 cm H_2_O and sustained for the next 30 seconds	Marginal improvement in oxygenation Deterioration of cerebral hemodynamics
Patroniti *et al.*[20]	2002	CPAP of 20% higher than peak airway pressure for 3–5 seconds every minute on pressure support ventilation PSV	Improvement in oxygenation Improved lung compliance
Grasso *et al.*[22]	2002	CPAP of 40 cm H_2_O for 40 seconds	Improved oxygenation noted only in early ARDS and in patients with low lung elastance
ARDSNet[32]	2003	CPAP of 35–45 cm H_2_O for 30 seconds	Effects of RM were inconsistent and transient
Constantin *et al.*[21]	2008	Comparative study between CPAP of 40 cm H_2_O for 40 sec and e-sigh by PEEP of 10 cm H_2_O above lower inflexion point on P–V curve for 15 minutes	Both maneuvers improved oxygenation at 5 and 60 minutes Drop in systolic pressure <70 mm Hg on two occasions in the CPAP group No significant drop in BP during e-sigh

## Monitoring of the efficacy of recruitment maneuvers

As more alveoli get recruited, the end-expiratory lung volume increases and arterial oxygenation would also be expected to rise. A practical measure of alveolar recruitment would be to monitor arterial blood gases. Malbouisson *et al.* showed a significant correlation between PEEP-induced alveolar recruitment and improved arterial oxygenation when recruitment was assessed by the volume of gas entering nonaerated or poorly aerated areas of the lung as seen on CT scans.[25] Apart from the obvious improvement in gas exchange, the efficacy of RMs can be assessed from the upward shift of the static volume-pressure curve[26] as well as the chest wall and lung elastance. Responders to RMs have been associated with a lower chest wall and lung elastance.[27]

## Who will benefit from recruitment maneuvers?

RMs have been studied extensively in patients with ARDS. Amato *et al.*, in their landmark paper, studied 53 patients with ARDS using a low tidal volume of 6mls/ kg on one arm combined with RMs and compared this with a tidal volume of 12mls/kg on the other arm. They found a significant mortality reduction in the low tidal volume group - 38 vs 71%.[28] Grasso *et al.* showed that patients with early ARDS had greater potential for alveolar recruitment compared to patients who had late ARDS.[22] They found a positive correlation between chest wall elastance and the duration of mechanical ventilation, possibly due to pleural effusions that accumulated over time. It is also likely that with time, fibroproliferative changes set in making the lungs more difficult to recruit. Pelosi *et al.* showed higher end-expiratory lung volumes, greater improvement in PO_2_, and lower venous admixture in extrapulmonary ARDS compared to pulmonary ARDS, suggesting greater potential for recruitment in the former condition.[29] In another study on patients with early ARDS, RMs in the form of sighs were carried out in the supine position and after turning prone. There was an improvement in PO_2_ after RMs in both supine and prone positions; however, the end-expiratory lung volume increased only with RMs, not with proning *per se*.[30] The authors suggested that the prone position might make the lungs topographically more favorable for RMs. Loss of end-expiratory lung volumes and fall in oxygen saturation could be prevented by the use of an RM during endotracheal suctioning, especially if a closed suction system is used.[31] In the ARDS Net trial that compared high versus low PEEP in ARDS patients, RMs were used in the first 80 patients randomly assigned to the high PEEP arm using a CPAP of 35–40cm of H_2_O for 30 seconds. However, this strategy was abandoned later on as the mean increase in arterial oxygenation was small and transient.[32] This might lend further support to the theory that if maximal recruitment has already been achieved with high levels of PEEP, further applications of RMs are unlikely to be of benefit. RMs have also been shown to prevent derecruitment during anesthesia and in postoperative cardiac surgical patients on mechanical ventilation.[33,34]

Gattinoni *et al.* studied 67 patients with ALI or ARDS using CT scans to assess recruitability.[15] Whole lung CT scans were done at an inspiratory plateau pressure of 45cm of H_2_O followed by PEEP levels of 5 and 15cm of H_2_O. The extent of recruitable lung was highly variable, with a mean of 13 ± 11% of the total lung weight. Progressive increase of airway pressure resulted in an increase of hyperinflated and normally aerated lung. More recruitable lung correlated with a higher fraction of nonaerated lung tissue, a lower P/F ratio, lower compliance, higher PCO_2_, greater shunt fraction, and increased mortality. Physiological variables such as improved P/F ratio, compliance, and reduced PCO_2_ predicted recruitability with a sensitivity of 71% and specificity of 59%.

A recent large randomized controlled trial addressed the value of higher levels of PEEP and RMs along with low tidal volume ventilation.[35] The authors targeted tidal volumes of 6mls/kg with an upper limit of plateau pressure of 30cm of H_2_O. On the study arm, they used a higher level PEEP based on the FiO_2_ according to a set protocol along with RMs using a sustained inflation of 40cm of H_2_O for 40 seconds. There was no difference in mortality between the two groups; however, there were significantly lower rates of refractory hypoxemia, death with refractory hypoxemia, and the use of predefined rescue therapies in the study arm.

## Possible harm from recruitment maneuvers

RMs would seem to be generally well tolerated. However, systemic hypotension is often seen, especially when a sustained inflation pressure is used.[22] A rise in intrathoracic pressure might increase the right ventricular afterload, compress the intrathoracic veins, and reduce the cardiac output. Besides, high intrapulmonary pressures can increase the right ventricular pressures leading to a shift of the interventricular septum towards the left ventricle. This can impair the left ventricular function and reduce cardiac output further. The lungs may also exert a compressive effect on the heart and impair cardiac compliance.[36] RMs may result in barotrauma, although the incidence is unknown. Bacterial translocation from the lung due to increased stretch during RMs is a theoretical concern.[37–39] However, Cakar *et al.*, in a rat model, showed that a sustained inflation RM of 45cm of H_2_O for 30 seconds at fifteen minute intervals did not result in bacterial translocation as evident on blood cultures. [40] Bein *et al.*, applied RMs to 11 patients with traumatic and nontraumatic brain lesions and found a significant rise in the intracranial pressure.[41] The mean arterial pressure dropped, thus reducing the cerebral perfusion pressure. The jugular venous oxygen saturation also fell significantly. There was only a modest improvement in arterial oxygen saturation. The authors recommended that RMs should not be performed in brain-injured patients.

## Summary

Recruitment maneuvers may prevent lung derecruitment and fall in oxygenation associated with low tidal volume ventilation strategies employed in ARDS. Patients who are already on optimal levels of PEEP are unlikely to benefit any further from RMs. Extrapulmonary ARDS is more likely to respond to RMs compared to pulmonary ARDS; consolidated lungs may not be recruitable. RMs are more likely to be successful during the early stages of ARDS, compared to the later stages when fibroproliferative changes set in. Over-recruitment can result in alveolar overdistension and diversion of blood away from ventilated to nonventilated areas, thus increasing the intrapulmonary shunt. Thus, RMs would always be a balancing act between opening of collapsed lung units and overdistending the already open units. Sustained inflation RMs may constitute an extreme increase of afterload to the right ventricle and result in significant hemodynamic compromise; hence, these may be less preferable to maneuvers involving intermittent high pressure. However, the optimal technique of recruitment is unclear and may vary depending on individual clinical circumstances. Although RMs have been clearly shown to improve gas exchange, there is no evidence that suggests improved morbidity or mortality. In fact, marginal improvements in gas exchange have not been shown to change clinical outcomes regardless of the strategy employed. On the other hand, the pursuit of better oxygenation using injurious ventilatory strategies is likely to cause harm. The primary role of RMs may be as rescue therapy in refractory hypoxia in patients with severe ARDS; their routine use in acute lung injury and ARDS may not be associated with benefit.
